# 

**Published:** 1851-10

**Authors:** 


### AMERICAN JOURNAL

OF

### DENTAL SCIENCE.

EDITED BY

CHAPIN A. HARRIS, M. D.

PROFESSOR OF THE PRINCIPLES AND PRACTICE OF DENTAL SURGERY IN THE BALTIMORE COLLEGE; MEMBER OF THE AMERICAN MEDICAL ASSOCIATION, ETC. ETC.;

AND

ALFRED A. BLANDY, M. D.

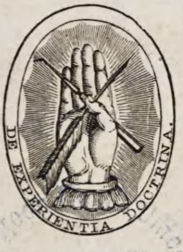

Surgeon General's Office LIBRARY Washington, D.C.

21657

NEW SERIES—VOLUME II.

PHILADELPHIA:
LINDSAY & BLAKISTON.
BALTIMORE:—ARMSTRONG & BERRY,
NEW ORLEANS:—JOHN BALL.
1851.-52

W
1
AM 45 M

JOHN W. WOODS, PRINTER,
BALTIMORE.

### TO READERS AND CORRESPONDENTS.

Communications intended for publication, and all letters relating to the business of the Journal, or containing remittances in money, should be directed to Drs. Harris and Blandy, Baltimore, Md.

### *Books and Pamphlets Received.*

1. *Elements of General and Pathological Anatomy*, presenting a view of the present state of knowledge in these branches of science. By David Craigie, M. D., F. R. S. E., Fellow of the Royal College of Physicians, Edinbugh, and Honorary Consulting Physician to the Royal Infirmary. Second Edition enlarged, Revised and Improved. Philadelphia: Lindsay and Blakiston, 1851. *From the Publishers.*

2. *The Microscopist*, or a complete Manual on the use of the Microscope, for Physicians, Students, and all lovers of Natural Science, with illustrations. By Joseph H. Wythes, M. D. Philadelphia: Lindsay and Blakiston, 1851. *From the Publishers.*

3. *Intermarriage*, or the mode in which, and the causes why, Health and Intellect results from certain unions, and Deformity, Disease and Insanity from others. Demonstrated by delineations of the structure and forms, and descriptions of the functions and capacities which each parent in every pair, bestows on children, in conformity with certain Natural laws, and by an account of corresponding effects in the breeding of animals, with eight illustrative drawings. By Alexander Walker. Philadelphia: Lindsay and Blakiston, 1851. *From the Publishers.*

4. *A plain Treatise on Dental Science and Practice.* By S. M. Shepherd, Dental Surgeon. Petersburg, Va., 1851. *From the Author.*

5. *A new Sign Language, for Deaf Mutes*, being a thesis for the Degree of Doctor in Medicine. Presented and sustained before the Medical Department of the Universiiy of Buffalo, February 25th, 1851. By Albert J. Myer, Buffalo, 1851.

6. *Seventh Annual Announcement of the Ohio College of Dental Surgery*, Session of 1851-2

7. *Annual Announcement of the Medical Department of the Washington University of Baltimore*, Session 1851-2.

8. *Annual Announcement of the Medical Faculty of McGill College, Montreal*, for Session 1851-2.

9. *Annual Announcement of the Medical Department of Pennsylvania College, Philadelphia*, Session 1851-2.

10. *Annual Announcement of Rush Medical College of Chicago, Illinois*, Session 1851-2.

11. Report on the Medical Department of the University of Pennsylvania, for the Session 1851-2. To the Alumni of the School. By the Medical Faculty. Philadelphia, 1851.

12. *Annual Announcement of the Baltimore College of Dental Surgeons*, Session 1851-2.

### *Medical and Dental Journals.*

13. *The Western Medico-Chirurgical Journal*, edited by J. F. Sanford, M. D., and Samuel G. Armor, M. D., Professors in Iowa State University, June, 1851. *In Exchange.*

14. *The American Journal of the Medical Sciences*, edited by Isaac Hays, M. D., Surgeon to *Wills Hospital*, &c., &c., July, 1851. *In Exchange.*

15. *The New York Dental Recorder.* Edited by C. C. Allen, M. D., Dentist. August, 1851. *In Exchange.*

16. *The Dental Register of the West.* Edited by James Taylor, M. D., D. D. S. Published by order of the Mississippi Valley Association of Dental Surgeons. July, 1851. *In Exchange.*

17. *The Dental News Letter.* July, 1851. *In Exchange.*

18. *The London Medical Gazette.* May, June and July, 1851. *In Exchange.*

19. *Boston Medical and Surgical Journal.* Edited by J. V. C. Smith, M. D. July, August and September, 1851. *In Exchange.*

20. *The British and Foreign Medico-Chirurgical Review.* July, 1851. *In Exchange.*

21. *The Ohio Medical and Surgical Journal.* Edited by Richard L. Howard, M. D. June and July, 1851. *In Exchange.*

22. *Quarterly Summary of the Transactions of the College of Physicians of Philadelphia*, from November 5th, 1850, to January 6th, 1851, inclusive, Vol. 1, No. 1. Philadelphia, 1851.

23. *The British American Medical and Physical Journal.* Edited by Archibald Hall, M. D. June, July and August, 1851. *In Exchange.*

24. *The New York Journal of Medicine and the Collateral Sciences.* Edited by S. S. Purple, M. D. June and July, 1851. *In Exchange.*

25. *The Medical Examiner.* Edited by F. G. Smith, M. D., and John Biddle, M. D. July, August and September, 1851. *In Exchange.*

26. *The Southern Medical and Surgical Journal.* Edited by J. P. Garvin, M. D. July and August. *In Exchange.*

27. *The New York Medical Gazette*, and *Journal of Health.* Edited by D. M. Reese, M. D., L. L. D. July, August and September. *In Exchange.*

28. *The New Jersey Medical Reporter, and Transactions of the New Jersey Medical Society.* Edited by Joseph Parish, M. D. August and September, 1851. *In Exchange.*

29. *The Western Lancet and Hospital Reporter.* Edited by L. M. Lawson, M. D., and George Mendenhall, M. D. July and August, 1851. *In Exchange.*

30. *The North Western Medical and Surgical Journal.* Edited by J. Evans, M. D., and E. G. Meek, A. M., M. D. July, 1851. *In Exchange.*

31. *The St. Louis Medical and Surgical Journal.* Edited by M. L. Linton, M. D., J. S. Moore, M. D., Wm. McPheeters, M. D., and J. B. Johnson, M. D. Not received since April.

32. *The Western Journal of Medicine and Surgery.* Edited by L. P. Yandell, M. D., and T. S. Bell, M. D. July and August, 1851. *In Exchange.*

### *To Readers and Correspondents.*

33. *The New Hampshire Journal of Medicine.* Edited by Edward H. Parker, A. M., M. D. July and August, 1851. *In Exchange.*

34. *The Charleston Medical Journal and Review.* Edited by D. J. Cain, M. D., and F. Peyrel Porcher, M. D. July, 1851. *In Exchange.*

35. *The Buffalo Medical Journal.* Edited by Austin Flint, M. D. July and August, 1851. In Exchange.

36. *The Provincial Medical and Surgical Journal.* Edited by W. H. Rankin, M. D., and J. H. Walsh, Esqr. May, June and July, 1851. In Exchange.

37. *The London Lancet,* Journal of British and Foreign, Surgical and Chemical Sciences, Chemistry, Literature, and News. Editor Thomas Wakeley, M. D., for London. Sub-Editors, J. H. Bennet, M. D., and T. Wakeley, M. R. C. S. E. July, August and September, 1851. In Exchange.

38. *The New York Register of Medicine and Pharmacy.* Edited by C. D. Griswold, M. D. July, August and September, 1851. In Exchange.

39. *The Northern Lancet and Gazette of Legal Medicine.* A monthly Journal of Medical and General Science, Criticism, and Medical Jurisprudence. Edited by Horace Nelson, M. D., and Frances J. D'Avignon, M. D. July, August and September, 1851. In Exchange.

40. *The American Journal of Insanity.* Published by the New York State Asylum, Utica. July, 1851. In Exchange.

41. *The Dublin Medical Press.* June, July and August, 1851. In Exchange.

42. *Monthly Journal of Medical Science.* Edited by William Robinson, M. D. Physician to the Royal Infirmary of Edinburgh. August, 1851. In Exchange.

Mucous Membrane of the Mouth, by Professor W. R. Handy, was received too late for publication in the present number of the Journal. It shall appear in the next. We are also compelled to omit several other interesting articles for want of room. They will appear in the January number.

Our correspondent desiring some information in regard to the use of the *Chloride of Zinc*, we refer to an article by Dr. Seabury, to be found in the first number of the Times.

To Subscribers.—We beg to remind our friends that the subscription to the Journal is due upon the reception of the first number, and we hope that its appearance will be considered the indication for prompt remittance.

To Delinquents.—Many are yet behind for the past year's subscription, and if such will enclose us ten dollars, for the past and the present year, we shall be very much gratified, and both be spared much annoyance. Our Terms are, payment in *advance*, or as we have just stated, after the receipt of the first number of each yearly volume. We have a great aversion to making out bills, differing widely in this respect, from those who supply the paper for the Journal, and our Printer.

*Omission.*—In the brief summary which we have given of the proceedings of the last meeting of the American Society of Dental Surgeons, we omitted a resolution offered by Dr. Townsend, and an address which he delivered on the occasion. They were not in our possession at the time we prepared the notice of the proceedings, and when they came into our hands, the printing of the Journal had progressed so far, that we had not sufficient room left for their insertion. They shall appear in the next number.

